# Supplementary material for: Feedbacks from the metabolic network to the genetic network reveal regulatory modules in E. coli and B. subtilis
Source: PLoS One. 2018 Oct 4;13(10):e0203311. doi: 10.1371/journal.pone.0203311 (PMC6171850; doi:10.1371/journal.pone.0203311)
Supplement: S7 Table — This file contains the details regarding randomization of a graph to create appropriate null model for comparison of results concerning the number and size of SCCs with those in the original graph. (PDF) [file pone.0203311.s007.pdf]

Supplementary Material for the manuscript titled "Feedbacks from the metabolic network to the genetic network reveal regulatory modules in *E. coli* and *B. subtilis*".

Santhust Kumar[1], Saurabh Mahajan[2], Sanjay Jain[1,3,*]

1 Department of Physics and Astrophysics, University of Delhi, Delhi 110007, India
2 National Centre for Biological Sciences, Bangalore, Karnataka 560065, India
3 Santa Fe Institute, 1399 Hyde Park Road, Santa Fe, NM 87501, USA

* E-mail: jain@physics.du.ac.in

Comparison of original networks with randomized versions of the networks

Result

		**Original Graph**		**Randomized graph**			
		**LSCC N**	**Number of SCCs**	**LSCC N, σ**	**Number of SCCs (range)**	**2nd largest SCC N, σ**	**Probability number of SCCs >=2**
E. coli	**Graph B**	378	20	480, 30	1-3	3, 1	0.10
	**Graph C**	97	28	285, 26	1-3	3, 1	0.13
B. subtilis	**Graph B**	85	9	225, 28	1-3	4, 2	0.18
	**Graph C**	13	15	125, 27	1-5	6, 6	0.24

N: number of nodes in the SCC; σ: standard deviation.

Randomization procedure

1000 successive instances of randomized graph were made starting with the original graph. For the generation of each randomization graph, a pair of edges (S1->T1; S2->T2) were randomly selected and checked for the following conditions before swapping the edges:

1. The edges S1->T2 and S2->T1 does not exist.
2. Additional conditions:
   i. [(S1 is gene AND T1 is gene) AND (S2 is gene AND T2 is gene)] **OR**
   ii. [(S1 is enzyme-gene AND T1 is metabolite) AND (S2 is enzyme-gene AND T2 is metabolite)] **OR**
   iii. [(S1 is metabolite AND T1 is tf-gene) AND (S2 is metabolite AND T2 is tf-gene)]

When the above conditions are satisfied, then the selected old edges are deleted and two new edges (S1->T2; S2->T1) are created. The process of randomly selecting a pair of edges, was repeated two times the total number of edges in the original graph. This procedure preserves the in- and out-degree distribution of the network. It also preserves the nature of different types of edges in the combined genetic and metabolic network.

Inference

From the table it is clear that the original graphs has larger number of SCCs compared to that expected from randomized graphs. Also, the size of the LSCC in original is less compared to that expected from randomized graphs. This is similar to the already observed fact that the LSCC in gene regulatory networks is smaller compared to that expected from randomized graphs using edge-swap procedure.
