# Supplementary material for: Feedbacks from the metabolic network to the genetic network reveal regulatory modules in E. coli and B. subtilis
Source: PLoS One. 2018 Oct 4;13(10):e0203311. doi: 10.1371/journal.pone.0203311 (PMC6171850; doi:10.1371/journal.pone.0203311)
Supplement: S1 Fig — This is a pdf file which contains the figure showing hierarchical structure of graph GC of E. coli. (PDF) [file pone.0203311.s008.pdf]

Supplementary Material for the manuscript titled "Feedbacks from the metabolic network to the genetic network reveal regulatory modules in *E. coli* and *B. subtilis*".

Santhust Kumar[1], Saurabh Mahajan[2], Sanjay Jain[1,3,*]

1 Department of Physics and Astrophysics, University of Delhi, Delhi 110007, India
2 National Centre for Biological Sciences, Bangalore, Karnataka 560065, India
3 Santa Fe Institute, 1399 Hyde Park Road, Santa Fe, NM 87501, USA

* E-mail: jain@physics.du.ac.in

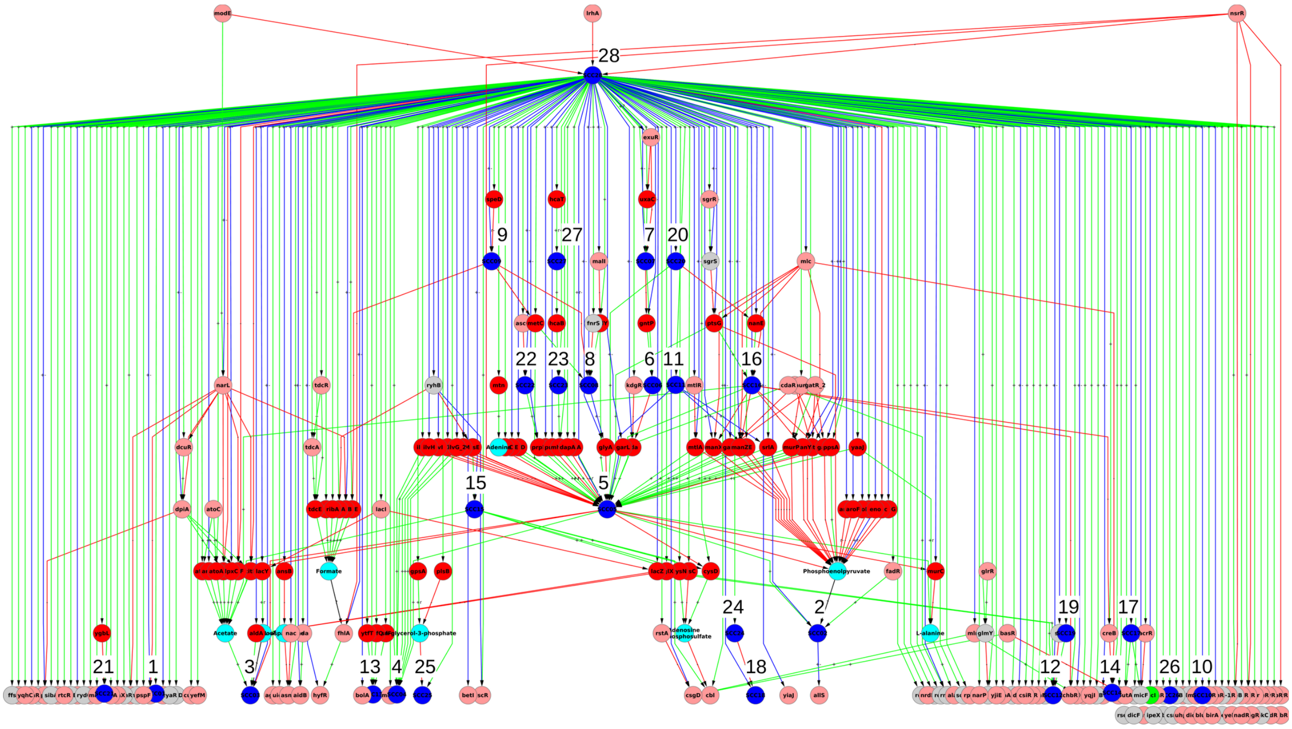

S1 Figure. The hierarchical structure of GRN of *E. coli*: Condensed version graph $\mathcal{G}_C$. The numbered blue nodes are SCCs whose detail is shown in Figs. 5 and 6 of the main text. Nodes and edges follow the same colour code as in Fig. 3 of the main text.
